# Associations between curve severity and revised Scoliosis Research Society-22 and scoliosis Japanese Questionnaire-27 scores in female patients with adolescent idiopathic scoliosis: a multicenter, cross-sectional study

**DOI:** 10.1186/s12891-021-04189-6

**Published:** 2021-03-29

**Authors:** Toru Doi, Kei Watanabe, Tokuhide Doi, Hirokazu Inoue, Ryo Sugawara, Yasuhisa Arai, Osamu Shirado, Ken Yamazaki, Koki Uno, Haruhisa Yanagida, So Kato, Yuki Taniguchi, Yoshitaka Matsubayashi, Yasushi Oshima, Sakae Tanaka, Katsushi Takeshita

**Affiliations:** 1 Department of Orthopaedic Surgery, The University of Tokyo, 7-3-1 Hongo, Bunkyo-ku, Tokyo, Japan; 2 Department of Orthopaedic Surgery, Niigata University Medical and Dental Hospital, 754, Ichibancho, Asahimachidori, Chuo-ku, Niigata-shi, Niigata, Japan; 3 Narita Tomisato Tokushuen, Geriatric Health Care Facility for the Elderly, 1-1-1 Hiyoshidai, Tomisato-shi, Chiba, Japan; 4 Department of Orthopaedic Surgery, Jichi Medical University, 3311-1 Yakushiji, Shimotsuke, Tochigi, Japan; 5 Tokyo Metropolitan Rehabilitation Hospital, 2-14-1 Tsutsumidori, Sumida-ku, Tokyo, Japan; 6 Department of Orthopaedic and Spinal Surgery, Aizu Medical Center, Fukushima Medical University, 21-2 Kawahigashimachitanisawa, Aizuwakamatsu, Fukushima, Japan; 7 Iwate Spinal Scoliosis Center, 103-1 Ogamayoshimizu, Takizawa, Iwate, Japan; 8 Department of Orthopaedic Surgery, National Hospital Organization Kobe Medical Center, 3-1-1 Nishiochiai, Suma-ku, Kobe, Hyogo Japan; 9 Fukuoka Children’s Hospital, 5-1-1 Kashiiteriha, Higashi-ku, Fukuoka, Japan

**Keywords:** Adolescent idiopathic scoliosis, Akaike information criterion, Cobb angle, Cutoff, Patient-reported outcome measure, SJ-27, SRS-22

## Abstract

**Background:**

Patient-reported outcome measures are widely utilized to assess health-related quality of life (HRQOL) in patients with adolescent idiopathic scoliosis (AIS). However, the association between HRQOL and curve severity is mostly unknown. The aim of this study is to clarify the association between HRQOL and curve severity, and to determine the optimal cutoff values of patient-reported outcomes for major curve severity in female patients with AIS.

**Methods:**

Female patients with AIS treated conservatively were recruited. The patients’ HRQOL outcomes were examined using the revised Scoliosis Research Society-22 (SRS-22r) and the Scoliosis Japanese Questionnaire-27 (SJ-27). The correlations of the SRS-22r and SJ-27 scores with the major Cobb angle were assessed using Spearman’s correlation coefficient analysis. The association between HRQOL issues in the SJ-27 and the major Cobb angle was evaluated by calculating Akaike’s Information Criterion (AIC). Furthermore, the optimal cutoff values of the SRS-22r and SJ-27 scores for the major Cobb angle were determined by AIC analysis.

**Results:**

The study cohort comprised 306 female patients with AIS. The SRS-22r and SJ-27 scores were significantly correlated with the major Cobb angle. Questions in the SJ-27 regarding discomfort when wearing clothes showed a lower AIC value in patients with severe scoliosis. The optimal cutoff values were a SRS-22r score of 3.2 for the discrimination of severe scoliosis (Cobb angle ≥48°), and a SJ-27 score of 32 for the discrimination of moderate scoliosis (Cobb angle ≥33°).

**Conclusion:**

Discomfort when wearing clothes was the most important HRQOL problem caused by severe scoliosis. The SRS-22r and SJ-27 scores are useful for the discrimination of clinical status in female patients with severe scoliosis or moderate scoliosis.

## Background

Adolescent idiopathic scoliosis (AIS) is the most common form of structural spinal deformity in childhood, which is defined as a lateral curvature of the spine of ≥10°. The prevalence of AIS is between 1 and 4% worldwide [1, 2]. AIS is more likely to progress to more severe deformity in females than males [3–8].

Therapeutic strategies for scoliosis include non-surgical and surgical treatments, which are commonly determined based on the spinal curve magnitude (major Cobb angle). Patients with AIS with mild scoliosis (Cobb angle < 25°) are usually treated via watchful waiting, followed by brace application for those with moderate scoliosis (Cobb angle between 25° and 45°) [2]. Surgery is generally recommended when the major curve progresses to an angle greater than 45° [2].

Previous studies reported that the spinal curve progression associated with AIS negatively affects patients’ health-related quality of life (HRQOL) [9–11]. Patients with untreated AIS reportedly have increased back pain, worse physical function, and psychosocial problems including lower self-image and greater depression than normal control subjects.

HRQOL is frequently measured using patient-reported outcome measures. The current gold standard for assessing HRQOL in patients with AIS is the revised version of the Scoliosis Research Society-22 questionnaire (SRS-22r) [12]. A 7-item Rasch-derived questionnaire (SRS-7) has been proposed as a short-form refinement of the SRS-22 [13]. The SRS-7 has been proved to be a valid and responsive functional outcome to measure HRQOL in patients with AIS [14]. The Japanese version of the SRS-22r has been established, and its reliability and validity have been proved [15]. However, the Japanese SRS-22r is thought to be at least partially suboptimal for Japanese patients with AIS because differences between Western and Japanese cultures. Therefore, the Scoliosis Japanese Questionnaire-27 (SJ-27) was developed as a novel patient-reported outcome measure for Japanese female patients with AIS, and its validity and reliability have been demonstrated [16]. The SJ-27 is characterized by the inclusion of questions regarding scoliosis-related discomfort when wearing clothes. To better understand the patients’ clinical status and determine the treatment method required at the appropriate time, it is necessary to clarify the association between HRQOL issues and major curve severity. Previous studies have used the SRS-22r to investigate the difference in HRQOL among curve-severity subgroups and reported significant differences in several domain and total scores in accordance with major curve severity [17–20]. However, it remains unclarified which HRQOL issue is significantly associated with major curve severity. Furthermore, to utilize the patient-reported outcome measures more effectively, it is crucial to determine the optimal cutoff values that indicate clinically significant spinal deformity in female patients with AIS. However, the optimal cutoff values of the SRS-22r or SJ-27 for scoliosis severity are still unknown.

The present study aimed to assess the association between each item of the SJ-27 and the major Cobb angle, and to clarify which HRQOL issue is significantly associated with major curve severity in female patients with AIS who are receiving conservative therapy. Furthermore, the present study sought to determine the optimal cutoff values of the SRS-22r and SJ-27 scores for the major Cobb angle in female patients with AIS.

## Methods

### Patients’ recruitment

This is a multicenter cross-sectional study. This study was conducted in accordance with the Declaration of Helsinki. The study protocol was approved by the institutional review board of the authors’ hospital (approval no. 3126). Written informed consent was obtained from all participants and their parents or legally authorized representatives. Female patients with AIS were recruited from outpatient clinics at 24 institutions in Japan between July 2009 and June 2011. Female patients with AIS were eligible for this study if they met the following inclusion criteria: (1) aged 10 to 18 years, (2) Cobb angle of the major curve ≥10°, (3) diagnosis of AIS confirmed by experienced spine specialists, (4) able to answer the Japanese questionnaire. As the present study aimed to examine HRQOL outcomes in female patients with AIS treated conservatively, patients with a history of spine surgery were excluded.

### Patient-reported outcome measures for female AIS patients

The patients’ HRQOL outcomes were assessed using the SRS-22r and the SJ-27. The SRS-22r covers four domains (function, pain, self-image, and mental health) consisting of five questions each, and one domain (satisfaction/dissatisfaction with management) consisting of two questions [12]. Each question has five verbal response alternatives ranging from 1 (worst) to 5 (best). The results of the SRS-22r are indicated as the mean score for each domain (minimum: 1 point, maximum: 5 points) and the total score (total sum of the domain divided by the number of items answered). The present study focused on determining the optimal cutoff value of the mean total SRS-22r score. The SRS-7 is a 7-item short form of the SRS-22 questionnaire (Q1, 4, 6, 10, 18, 19, and 20). SRS-7 raw scores were calculated from patient responses to corresponding SRS-22r items.

The SJ-27 was designed to cover a wide range of HRQOL issues among young female patients with AIS in Japan, including four questions regarding back pain (Q1–4), seven questions regarding discomfort while wearing clothes (Q5–10 and 27), four questions regarding physical activities (Q11, 15, 16, and 19), six questions regarding self-consciousness about scoliosis (Q12–14, 22, 25, and 26), and six questions related to psychological disorders such as depression (Q17, 18, 20, 21, 23, and 24) [16]. The SJ-27 consists of 27 items that are scored on a 5-point scale from no impairment (0 point) to severe impairment (4 points) and then added to yield a total score ranging from 0 to 108 points. A higher SJ-27 score indicates a worse HRQOL, which is opposite to the scoring method of the SRS-22r.

### Data collection and radiographic assessment

The following data were collected from the documents recorded by the attending physicians at each institution: age, body mass index, and AIS treatment history (observation, therapeutic exercise, and/or brace [multiple responses allowed]). Standing anteroposterior radiographs of the whole spine were performed at the time of the questionnaire administration. The Cobb method was used to measure the major curve angles.

### Statistical analysis

Data are expressed as mean (standard deviation). Spearman’s correlation coefficient analysis was used to assess the correlations of the SRS-22r, SRS-7, or SJ-27 scores with the major Cobb angle. Moreover, we performed the multivariate linear regression analysis in consideration of the impact of treatment methods on the SRS-22r and SJ-27 scores. Variables such as age, major Cobb angle, and treatment (therapeutic exercise or brace treatment) were subjected to multivariate analysis as possible associated factors with each HRQOL outcome. Akaike’s Information Criterion (AIC) analysis was used to determine the optimal cutoff values of the SRS-22r and SJ-27 scores for the Cobb angle of the major curve. AIC analysis is widely used to compare different models with the same data, with a lower AIC value indicating a more reliable model for predicting outcomes. The AIC method has been previously utilized to determine cutoff values in various clinical fields [21–23]. AIC values were calculated for all combinations between each cutoff point of the total SRS-22r or SJ-27 score and each cutoff point of the major Cobb angle. The lowest AIC value indicated the optimal cutoff score of the SRS-22r or SJ-27 for predicting scoliosis severity. The same statistical method was used to examine the association between each item of the SJ-27 and the major Cobb angle in female patients with AIS. The 5-point scales (0–4 points) of each of the 27 questions were classified as 0 or 1–4 points, 0–1 or 2–4 points, 0–2 or 3–4 points, and 0–3 or 4 points. AIC values were calculated for all combinations between each dichotomization and each cutoff point of the major Cobb angle. A lower AIC value indicated that a question was more relevant to HRQOL issues caused by scoliosis (major Cobb angle) in female patients with AIS. A *p* value < 0.05 was considered statistically significant. All data were analyzed using IBM SPSS Statistics, version 23 (IBM Corp., Armonk, NY, USA).

## Results

### Patient characteristics

A total of 405 female patients with AIS were recruited. Of these patients, 21 patients > 18 years of age were excluded, and 78 patients were excluded because of a history of surgery for scoliosis. Finally, 306 female patients with AIS were enrolled in this study. Table 1 presents the demographic characteristics of the 306 included patients. The mean age was 14.2 (1.9) years, mean body mass index was 18.4 (2.3), and mean Cobb angle was 32.9 (11.6)°. Brace treatment was applied to 160 patients (52.3%).

**Table 1:** Demographic data of 306 female patietns with adolescent idiopathic scoliosis

Patients characteristics (***n*** = 306)	Mean (SD)
Age, years	14.2 (1.9)
BMI, kg/m^2^	18.4 (2.3)
Cobb angles, degree	32.9 (11.6)
Treatment method (multiple choices allowed)	
Observation, n	119
Therapeutic exercise, n	28
Brace, n	160

*Abbreviations: BMI*, body mass index; *SD*, standard deviation

### Correlations between HRQOL outcomes and major curve severity

Two-hundred-and-eighty-nine patients were included in the analysis of the association between the SRS-22r scores and the major Cobb angle (17 patients were excluded due to a lack of SRS-22r data or radiographic data), whereas 296 patients were included in the analysis of the association between the SJ-27 scores and the major Cobb angle (10 patients were excluded due to a lack of required data). Spearman’s correlation coefficient analysis showed that significant negative or positive correlations were observed between the SRS-22r score and the major Cobb angle (*ρ* = − 0.315, *p* <  0.001), between the SRS-7 score and the major Cobb angle (*ρ* = − 0.322, *p* <  0.001), and between the SJ-27 score and the major Cobb angle (*ρ* = 0.332, *p* <  0.001). Multivariate linear regression analysis yielded that the major Cobb angle and age were significantly associated with both the SRS-22r (major Cobb angle, *β* = − 0.352, *p* <  0.001; age, *β* = − 0.187, *p* = 0.001) and the SJ-27 (major Cobb angle, *β* = 0.314, *p* <  0.001; age, *β* = 0.241, *p* <  0.001) (Table 2). However, main treatment methods for AIS such as brace treatment or observation had no significant association with both the SRS-22r and the SJ-27 scores (Table 2).

**Table 2:** Multivariate linear regression analysis for SRS-22r or SJ-27 score

	SRS-22r			SJ-27		
Variables	*β*	P value	95% CI	*β*	P value	95% CI
Cobb angle	−0.352	< 0.001	−0.015 to − 0.008	0.314	< 0.001	0.296 to 0.609
Age	−0.187	0.001	−0.059 to − 0.016	0.241	< 0.001	1.198 to 3.103
Treatment	−0.010	0.182	−0.093 to 0.077	0.081	0.145	−0.942 to 6.377

*β,* standardized coefficients

*Abbreviations: SJ-27,* Scoliosis Japanese Questionnaire-27; *SRS-22r,* revised Scoliosis Research Society-22; *95% CI,* 95% confidence interval

### Association between HRQOL issues in the SJ-27 and major curve severity

**Fig. 1:**
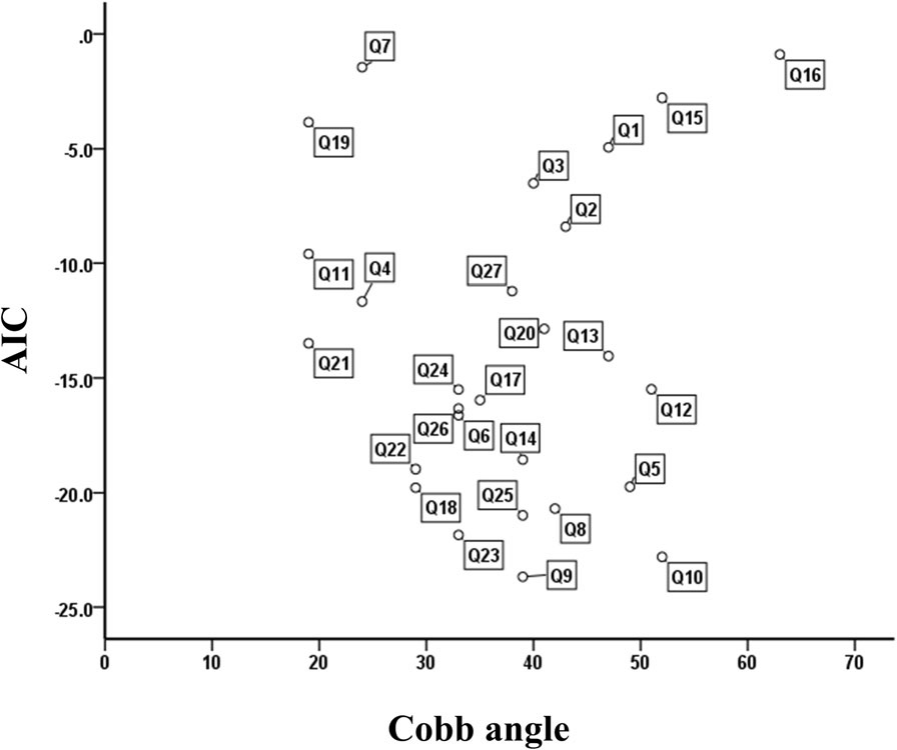
Akaike’s Information Criterion of each of the 27 questions in the Scoliosis Japanese questionnaire-27 for the major Cobb angle. *Abbreviation*: *AIC* Akaike’s Information Criterion

Figure 1 shows the AIC values and corresponding major Cobb angles for each question in the SJ-27 (Q1–27). Q9 (“To what extent are you bothered by the slipping of bra or camisole straps from your shoulders?”) had the minimum AIC value (− 23.67) when the major Cobb angle was 39°. Furthermore, other questions related to discomfort when wearing clothes, especially on the upper body (Q5, Q8, and Q10), showed relatively lower AIC values (− 19.74, − 20.69, and − 22.80, respectively) when the major Cobb angle ranged from 42° to 52°. In contrast, Q6, Q7, and Q27 related to discomfort when wearing lower body garments or socks and holding bags had relatively higher AIC values (− 16.63, − 1.45, and − 11.20, respectively) compared with the abovementioned questions. These results suggest that discomfort when wearing clothes on the upper body was considered an important HRQOL issue caused by relatively severe scoliosis (Cobb angle 40° to 50°) in young female patients with AIS.

Q18, Q22, Q23, and Q25 regarding the psychological problems caused by spinal deformity had relatively smaller AIC values (− 19.78, − 18.98, − 21.84, and − 20.98, respectively) when the major Cobb angle ranged from 29° to 39° (Fig. 1). These results indicate that the psychological problems such as depression or self-consciousness related to scoliosis were considered crucial HRQOL issues caused by moderate scoliosis (Cobb angle 30° to 40°) in young female patients with AIS.

### Cutoff value of the SRS-22r score for the cobb angle

**Fig. 2:**
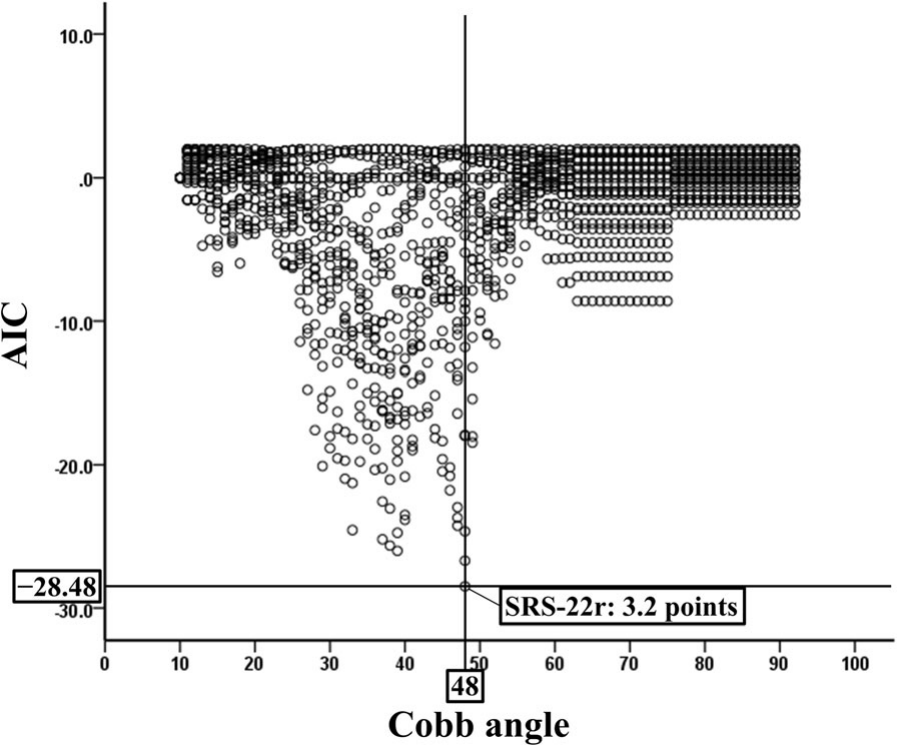
Akaike’s Information Criterion of each revised Scoliosis Research Society-22 cutoff value for the major Cobb angle. *Abbreviations*: *AIC* Akaike’s Information Criterion; *SRS-22r* revised Scoliosis Research Society-22

Figure 2 shows that the minimum AIC value (− 28.48) was observed for a SRS-22r score of 3.2 when the major Cobb angle was 48°. This result indicates that a mean total SRS-22r score of 3.2 was the optimal cutoff value for the discrimination of severe scoliosis (Cobb angle ≥48°) in female patients with AIS.

### Cutoff value of the SJ-27 score for the cobb angle

**Fig. 3:**
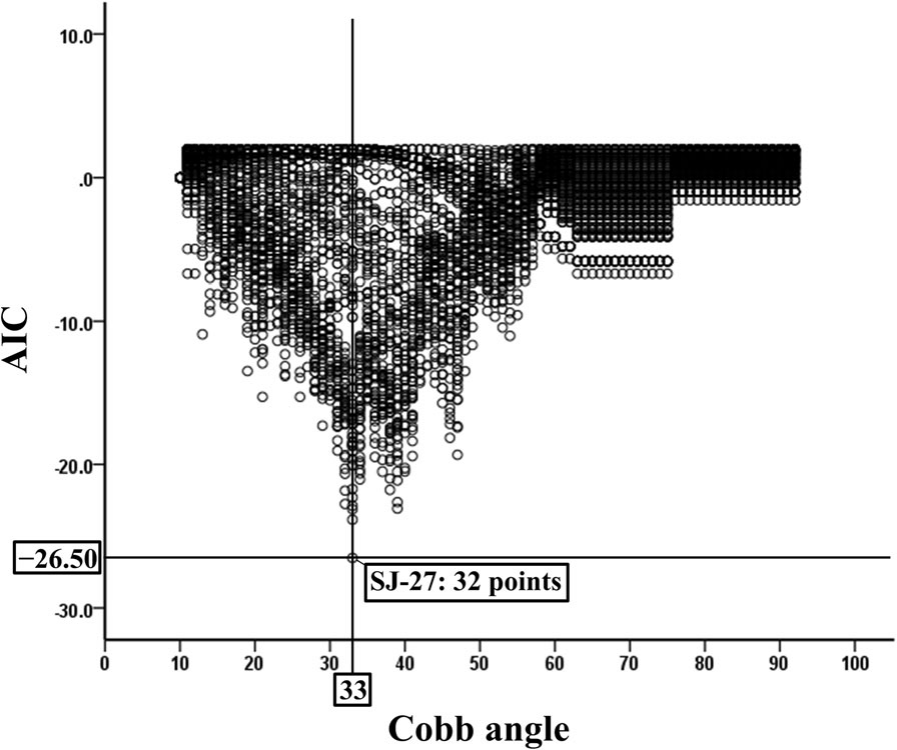
Akaike’s Information Criterion of each Scoliosis Japanese Questionnaire-27 cutoff value for the major Cobb angle. *Abbreviations*: *AIC* Akaike’s Information Criterion; *SJ-27* Scoliosis Japanese Questionnaire-27

Figure 3 shows that a SJ-27 score of 32 had the lowest AIC value (− 26.50) when the major Cobb angle was 33°. This result indicates that a total SJ-27 score of 32 was the optimal cutoff value for the discrimination of moderate scoliosis (Cobb angle ≥33°) in female patients with AIS.

## Discussion

Our study revealed the following important findings. First, the SRS-22r, SRS-7, and SJ-27 scores were significantly correlated with the major Cobb angle. Second, discomfort when wearing clothes on the upper body is an important HRQOL issue caused by severe scoliosis (Cobb angle 40° to 50°) in female patients with AIS. Third, psychological problems such as depression or self-consciousness related to scoliosis were crucial HRQOL issues caused by moderate scoliosis (Cobb angle 30° to 40°). Finally, the optimal cutoff values were a SRS-22r score of 3.2 for the discrimination of severe scoliosis (Cobb angle ≥48°), and a SJ-27 score of 32 for the discrimination of moderate scoliosis (Cobb angle ≥33°).

Significant correlations between each outcome score and the major Cobb angle indicate that the SRS-22r, SRS-7, and SJ-27 scores may reflect the clinical HRQOL status in association with major curve severity. Similarly, Parent et al. reported a significant negative correlation between the total SRS-22 score and the Cobb angle (*ρ* = − 0.24, *p* < 0.05) [19].

AIC analysis showed that the SJ-27 questions related to discomfort when wearing clothes were associated with relatively severe scoliosis. Shoulder height asymmetry or a shift of the trunk due to severe scoliosis may negatively affect the feelings or behaviors of female patients with AIS when wearing clothes on the upper body. Given that surgical treatment is generally recommended for severe scoliosis, [2] discomfort when wearing clothes on the upper body might indicate the need for surgical treatment.

AIC analysis also demonstrated that psychological problems related to scoliosis were associated with moderate scoliosis. This implies that female patients with AIS may be more likely to recognize their deformed appearance when the curve progresses to moderate scoliosis (Cobb angle 30° to 40°). Given that treatment with a brace is generally recommended for patients with moderate scoliosis, [2] psychological problems caused by scoliosis might indicate the need for brace application. These findings may prompt clinicians to assess patients’ HRQOL corresponding to the major curve severity and aid in the determination of the adequate treatment method for female patients with AIS.

Previous studies have demonstrated the discriminative validity of the SRS-22 among curve-severity subgroups differentiated by an arbitrarily determined Cobb angle [17–20]. Unlike in previous studies, the present study obtained the optimal cutoff values of the SRS-22r or SJ-27 by calculating AIC values for all combinations between each cutoff point of each total outcome score and each cutoff point of the major Cobb angle by every 1 point or 1°. The present results showed that the SJ-27 had an optimal cutoff value of 32 points for a lower cutoff of Cobb angle (33°) compared with the SRS-22r (optimal cutoff value of 3.2 points for a Cobb angle of 48°). These results imply that the SRS-22r may be suitable for the detection of patients with severe scoliosis who may need surgical treatment, while the SJ-27 may be suitable for the detection of patients with moderate scoliosis who may need treatment with a brace. Using these cutoff scores may help clinicians to classify female patients with AIS into clinical severity groups (mild, moderate, and severe) and determine the appropriate therapeutic intervention.

The present study has several limitations. First, the study cohort included patients who received therapeutic exercise or brace treatment. However, given that multivariate regression analysis showed no significant association between main treatment methods for AIS such as therapeutic exercise or brace treatment and each HRQOL outcome, main treatment methods for AIS had little effect on the study results. Second, the study had no healthy control subjects. A comparison study between patients with AIS and controls may provide more meaningful results. Third, other confounding factors that may have affected the study results were not evaluated, including shoulder imbalance, pelvic obliquity, skeletal maturity, sport activity, or socioeconomic environment. Further study is needed to evaluate the effect of various factors on the HRQOL outcomes in female patients with AIS. Fourth, the effect of therapeutic intervention (such as surgery) on HRQOL outcomes was not examined. The patient-reported outcome measures (especially discomfort when wearing clothes) will be used to assess the postoperative alterations in HRQOL outcomes in future studies. Fifth, this study did not investigate the curve type, which may affect patients’ HRQOL issues including discomfort when wearing clothes on the upper body. Further investigation will be considered to assess the influence of curve type on HRQOL outcomes in patients with AIS. Sixth, we did not examine the cutoff values of other HRQOL outcomes for major Cobb angle. The SRS-7 score showed a significant correlation with major Cobb angle in female patients with AIS. In future work, we hope to assess the cutoff value of the SRS-7, a simplified version of the SRS-22r, for major Cobb angle. Finally, this study included only Japanese female patients with AIS. We expect to examine the association between HRQOL outcomes and curve severity in female patients with AIS in other countries.

## Conclusion

Our study demonstrated that discomfort when wearing clothes on the upper body was the most important HRQOL issue caused by severe scoliosis in female patients with AIS. The optimal cutoff values of the SRS-22r and the SJ-27 are useful for the discrimination of clinical status in female patients with severe or moderate AIS.

## Data Availability

The data in current study is available from corresponding author on reasonable request.

## Abbreviations

AIS: Adolescent idiopathic scoliosis
HRQOL: Health-related quality of life
SRS-22r: Revised version of the Scoliosis Research Society-22
SJ-27: Scoliosis Japanese Questionnaire-27
AIC: Akaike’s Information Criterion

## References

[1] Cheng JC, Castelein RM, Chu WC, Danielsson AJ, Dobbs MB, Grivas TB, Gurnett CA, Luk KD, Moreau A, Newton PO, Stokes IA, Weinstein SL, Burwell RG (2015). Adolescent idiopathic scoliosis. Nat Rev Dis Primers.

[2] Weinstein SL, Dolan LA, Cheng JC, Danielsson A, Morcuende JA (2008). Adolescent idiopathic scoliosis. Lancet.

[3] Bunnell WP (2005). Selective screening for scoliosis. Clin Orthop Relat Res.

[4] Lonstein JE (1994). Adolescent idiopathic scoliosis. Lancet.

[5] Lonstein JE, Carlson JM (1984). The prediction of curve progression in untreated idiopathic scoliosis during growth. J Bone Joint Surg Am.

[6] Reamy BV, Slakey JB (2001). Adolescent idiopathic scoliosis: review and current concepts. Am Fam Physician.

[7] Roach JW (1999). Adolescent idiopathic scoliosis. Orthop Clin North Am.

[8] Tan KJ, Moe MM, Vaithinathan R, Wong HK (2009). Curve progression in idiopathic scoliosis: follow-up study to skeletal maturity. Spine (Phila Pa 1976).

[9] Danielsson AJ, Wiklund I, Pehrsson K, Nachemson AL (2001). Health-related quality of life in patients with adolescent idiopathic scoliosis: a matched follow-up at least 20 years after treatment with brace or surgery. Eur Spine J.

[10] Freidel K, Petermann F, Reichel D, Steiner A, Warschburger P, Weiss HR (2002). Quality of life in women with idiopathic scoliosis. Spine (Phila Pa 1976).

[11] Tones M, Moss N, Polly DW (2006). A review of quality of life and psychosocial issues in scoliosis. Spine (Phila Pa 1976).

[12] Asher MA, Lai SM, Glattes RC, Burton DC, Alanay A, Bago J (2006). Refinement of the SRS-22 health-related quality of life questionnaire function domain. Spine (Phila Pa 1976).

[13] Caronni A, Zaina F, Negrini S (2014). Improving the measurement of health-related quality of life in adolescent with idiopathic scoliosis: the SRS-7, a Rasch-developed short form of the SRS-22 questionnaire. Res Dev Disabil.

[14] Jain A, Sponseller PD, Negrini S, Newton PO, Cahill PJ, Bastrom TP, Marks MC, Harms Study G (2015). SRS-7: a valid, responsive, linear, and Unidimensional functional outcome measure for operatively treated patients with AIS. Spine (Phila Pa 1976).

[15] Hashimoto H, Sase T, Arai Y, Maruyama T, Isobe K, Shouno Y (2007). Validation of a Japanese version of the Scoliosis Research Society-22 patient questionnaire among idiopathic scoliosis patients in Japan. Spine (Phila Pa 1976).

[16] Doi T, Inoue H, Arai Y, Shirado O, Doi T, Yamazaki K, Uno K, Yanagida H, Takeshita K (2018). Reliability and validity of a novel quality of life questionnaire for female patients with adolescent idiopathic scoliosis: scoliosis Japanese Questionnaire-27: a multicenter, cross-sectional study. BMC Musculoskelet Disord.

[17] Asher M, Min Lai S, Burton D, Manna B (2003). Discrimination validity of the scoliosis research society-22 patient questionnaire: relationship to idiopathic scoliosis curve pattern and curve size. Spine (Phila Pa 1976).

[18] Berliner JL, Verma K, Lonner BS, Penn PU, Bharucha NJ (2013). Discriminative validity of the Scoliosis Research Society 22 questionnaire among five curve-severity subgroups of adolescents with idiopathic scoliosis. Spine J.

[19] Parent EC, Hill D, Mahood J, Moreau M, Raso J, Lou E (2009). Discriminative and predictive validity of the scoliosis research society-22 questionnaire in management and curve-severity subgroups of adolescents with idiopathic scoliosis. Spine (Phila Pa 1976).

[20] Rainoldi L, Zaina F, Villafane JH, Donzelli S, Negrini S (2015). Quality of life in normal and idiopathic scoliosis adolescents before diagnosis: reference values and discriminative validity of the SRS-22. A cross-sectional study of 1,205 pupils. Spine J.

[21] Doi T, Doi T, Kawamura N, Matsui T, Komiya A, Tei Z, Niitsuma G, Kunogi J (2016). The usefulness of neutrophil CD64 expression for diagnosing infection after orthopaedic surgery in dialysis patients. J Orthop Sci.

[22] Kim SG, Nagao M, Nozawa M, Doi T (2019). Optimal cutoff score for patient-reported outcome measures after anterior cruciate ligament reconstruction using load-displacement curve analysis. J Orthop Surg (Hong Kong).

[23] Shibahashi K, Doi T, Tanaka S, Hoda H, Chikuda H, Sawada Y, Takasu Y, Chiba K, Nozaki T, Hamabe Y, Ogata T (2016). The serum phosphorylated Neurofilament heavy subunit as a predictive marker for outcome in adult patients after traumatic brain injury. J Neurotrauma.

